# Trifolirhizin induces autophagy-dependent apoptosis in colon cancer via AMPK/mTOR signaling

**DOI:** 10.1038/s41392-020-00281-w

**Published:** 2020-08-27

**Authors:** Dongdong Sun, Weiwei Tao, Feng Zhang, Weixing Shen, Jiani Tan, Liu Li, Qinghai Meng, Yugen Chen, Ye Yang, Haibo Cheng

**Affiliations:** 1 Collaborative Innovation Center of Jiangsu Province of Cancer Prevention and Treatment of Chinese Medicine, 210023 Nanjing, China; 2 School of Integrated Chinese and Western Medicine, Nanjing University of Chinese Medicine, 210023 Nanjing, China; 3 Jiangsu Key Laboratory for TCM Formulae Research, 210023 Nanjing, China; 4 Kunshan TCM Hospital Affiliated to Nanjing University of Chinese Medicine, 215300 Kunshan, China; 5 The First School of Clinical Medicine, The Affiliated Hospital of Nanjing University of Chinese Medicine, 210029 Nanjing, China

**Keywords:** Gastrointestinal cancer, Cancer metabolism

**Dear Editor**,

Colorectal cancer (CRC) is one of the most frequent diseases with high mortality around the world. Conventional treatments of CRC remain unsatisfactory due to the increasing recurrence rate and adverse reactions including neutropenia, drug resistance, etc. Recently, autophagy has been shown to be involved in regulating cancer development and progression by regulating apoptosis through pro-apoptosis proteins including caspases, in addition to being a potential target for cancer therapeutic intervention.^1^ Trifolirhizin (Supplementary Fig. S1a) is a natural flavonoid glycosides isolated from *Sophora flavescens* as well as a bio-active constituent of Xian-Lian-Ke-Li, a commercial traditional Chinese medicine for the cancer prevention. Numerous evidence manifested that trifolirhizin inhibited proliferative activity in melanoma B16 cell, lung cancer H23 cell, human ovarian A2780 cell, and human gastric cancer cell MKN45.^2^ However, its pharmacological effect and mechanism on CRC remain elusive. Herein, we evaluated the effect of trifolirhizin on autophagy and apoptosis on CRC as well as its related mechanisms, in order to provide evidence to develop a potential agent with less adverse effect in treating CRC.

First, we detected whether trifolirhizin could induce autophagy in two CRC cell lines, HCT116 and SW620 cells. When treated with trifolirhizin, the cell morphological changes were seen (Supplementary Fig. S1b) and autophagy was preliminarily confirmed in LC-3 and p62 by immuno-blotting assay of two autophagy marker proteins, LC-3 and p62/SQSTM-1 (Fig. 1c and Supplementary Fig. S1d, e). Dose- and time-related accumulation of LC3B-I and LC3B-II were observed in trifolirhizin-treated HCT116 and SW620 cells, and the expression ratio of LC3B-II/I was also increased, whereas the protein expression of SQSTM-1 was downregulated. LC3B, SQSTM1, and poly-ubiquitination level in the detergent-soluble and detergent-insoluble fractions were further analyzed. The content of SQSTM1 decreased and LC3B-II and poly-ubiquitin aggregated in the detergent-insoluble fractions after trifolirhizin treatment. Furthermore, the visualization and quantification of autophagic vacuoles (double-membrane compartments containing lamellar structures) were achieved by transmission electron microscopy (Fig. 1b and Supplementary Fig. S1g). The Ad-mCherry-GFP-LC3B fluorescent assay was used to monitor the autophagy flux; trifolirhizin treatment increased both green and red dots, which represented autophagosomes and autophagolysosomes, respectively (Supplementary Fig. S1f). These results displayed that trifolirhizin accelerated autophagy flux in CRC cells.

**Fig. 1:**
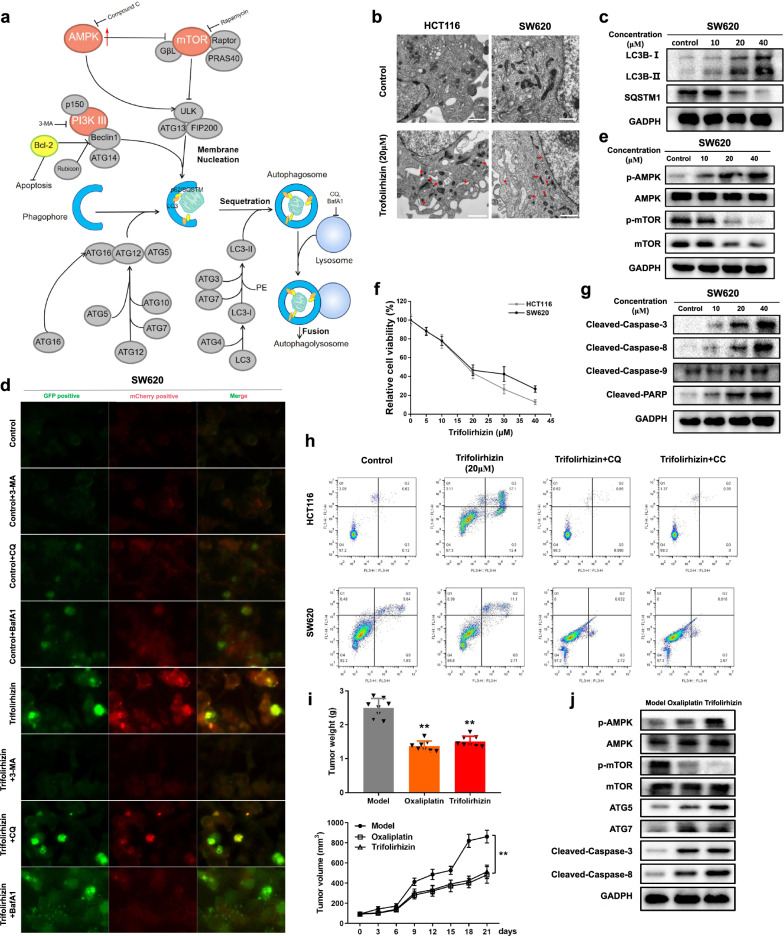
Trifolirhizin induced autophagy-mediated CRC apoptosis in vitro and in vivo. **a** The diagram of autophagy mechanism. **b** Images were taken by scanning electron microscope after cells were treated with trifolirhizin (20 μM) for 6 h. Arrow indicates autophagosome including the lamellar structure. (Scale bar: 1 μm). **c** Immuno-blotting assay of LC3B-I, LC3B-II, and SQSTM1 protein expression. GADPH was used as the loading control (*n* = 3). **d** Cells were treated with 20 μM trifolirhizin in the presence or absence of 10 mM 3-MA or 20 μM CQ or 10 nM BafA1. Cells were mixed with Ad-mCherry-GFP-LC3B and then incubated with trifolirhizin for 6 h, and the mCherry (red) and GFP (green) were analyzed by a laser scanning confocal microscope (LSCM) (*n* = 3). **e** HCT116 and SW620 cells were treated with either different concentrations or time durations of trifolirhizin. Western blot assay of p-AMPK, AMPK, p-mTOR, and mTOR protein expression (*n* = 3). **f** The cells were exposed to different concentrations of trifolirhizin for 48 h, and the cell viability was measured by CCK-8 assay (*n* = 10). **g** Expression levels of cleaved caspase-9, cleaved caspase-8, cleaved caspase-3, cleaved PARP, and cytochrome c were determined by western blot (*n* = 3). **h** Early apoptosis of the cell was analyzed by flow cytometry with AnnexinV-FITC/PI staining (*n* = 6). **i** Tumor-bearing mice were treated with trifolirhizin, Oxaliplatin (positive drug), or with vehicle control. Tumor size was measured every 3 days, and tumors were harvested and weighed after 4 weeks of treatment (*n* = 8). **j** Western blot assays of p-AMPK, AMPK, p-mTOR, mTOR, Atg5, Atg7, cleaved-caspase-8, and cleaved-caspase-3 expression in tumor section (*n* = 3). **P < 0.01 versus model

These aforementioned observations are generally but not always indicators for the cellular autophagic activity. It is essential to analyze autophagic flux by adding the autophagy inhibitors. In the present study, 3-methyladenine (3-MA), chloroquine (CQ), and Bafilomycin A1 (BafA1) were employed. 3-MA suppresses autophagy by preventing autophagosome formation through the blockage of type III phosphatidylinositol 3-kinases; CQ is an inhibitor for the fusion of autophagosome with lysosome; BafA1 is a V-ATPase inhibitor that blocks autophagosome and lysosome fusion by increasing pH value of lysosome. We found that, to different degrees, autophagy flux was impaired after co-treatment with trifolirhizin and autophagy inhibitors (Fig. 1d and Supplementary Fig. S2).

Various molecules have been implicated in the upregulation and downregulation of autophagy; we focused on one of the classical regulatory pathways, AMPK/mammalian target of rapamycin (mTOR) signaling. Phosphorylated mTOR inhibits autophagy, while the activation of AMPK, the AMP-dependent protein kinase that adjusts energy metabolism in eukaryotic cells, may induce the dephosphorylation of mTOR and thus modulates cell proliferation and metabolism.^3^ In the present study, we found that trifolirhizin treatment exacerbated the phosphorylation of AMPK, while it blocked the phosphorylation of mTOR in CRC cells (Fig. 1e and Supplementary Fig. S3a, b). AMPK activation was essential for trifolirhizin-induced autophagy because both of them were attenuated when AMPK was knocked down by its specific inhibitor or small interfering RNA (siRNA; Supplementary Fig. S3c–f). In contrast, mTOR inhibitor rapamycin could simulate and enhance the effect of trifolirhizin in inducing autophagy (Supplementary Fig. S3g, h).

Next, we analyzed the apoptotic pathway as apoptosis often occurs with autophagy. First, it turned out that the short-term treatment with trifolirhizin significantly reduced the cell viabilities of the two examined cells in a dose-dependent manner (Fig. 1f). Furthermore, long-term growth inhibitory effect of trifolirhizin was confirmed by colony formation assay (Supplementary Fig. S4a). Flow cytometric analysis and terminal deoxynucleotidyl transferase-mediated dUTP-fluorescein nick end labeling (TUNEL) staining showed that trifolirhizin induced both early and late apoptosis of HCT116 and SW620 cells (Supplementary Fig. S4b, c). We briefly examined the mechanism of trifolirhizin-induced apoptosis. The intrinsic apoptosis pathway is activated by the release of cytochrome c from mitochondria. Cytochrome c combines with the caspase-activating protein Apaf-1 and induces the binding of Apaf-1 to pro-caspase-9, which promotes the activation of caspase and then directly stimulates the effector caspase to accelerate apoptosis.^4^ However, according to our study, the expression of cleaved caspase-9 and cytochrome c remained unchanged after trifolirhizin treatment (Fig. 1g and Supplementary Fig. S4d, e), suggesting that it may induce apoptosis through the extrinsic pathway, which further evidenced by the elevation of cleaved poly ADP-ribose polymerase, cleaved caspase-3, and cleaved caspase-8. Its mechanism might be attributed to the activation of death receptors that trigger the FADD recruitment, thus aggregating caspase-8 and promoting its autoprocessing and activation.^5^ The pan-caspase inhibitor Z-VAD-FMK obviously decreased the cytotoxicity of trifolirhizin against CRC cells (Supplementary Fig. S4f–g), proving that trifolirhizin induced apoptosis in HCT116 and SW620 cells in a caspase-mediated way.

Furthermore, the relationship between apoptosis and autophagy caused by trifolirhizin was explored. CQ, Compound C, and ATG5 siRNA were applied to inhibit late autophagy, AMPK signaling, and early autophagy, respectively. Trifolirhizin-induced apoptosis was sharply reversed by inhibiting AMPK activation using its inhibitor, Compound C. Inhibition of autophagy by CQ and ATG-5 siRNA achieved similar results (Fig. 1h and Supplementary Fig. S5a–g). Autophagy-associated apoptosis was marked by Lamp1 and TUNEL double staining (Supplementary Fig. S5h). As the concentration of trifolirhizin increase, the fluorescence of TUNEL and LAMP1 puncta intensified simultaneously in two CRC cell lines, exhibiting the autophagy-associated cell death was induced by trifolirhizin.

We established a CRC model using C57BL/6 mice to explore the role of trifolirhizin in vivo. Oxaliplatin (5 mg/kg) was chosen as a positive control. Twenty-one days later, xenograft tumors treated with trifolirhizin (10 mg/kg) were much smaller than the model group (Fig. 1i), showing that trifolirhizin could effectively suppress tumor growth. Prominent necrosis and apoptosis of tumor section was visualized in the treatment group by hematoxylin and eosin (H&E) and TUNEL staining, demonstrating that trifolirhizin could destruct tumor cells (Supplementary Fig. S6c, d). Immuno-staining assay of tumor sections showed that trifolirhizin treatment increased the numbers of p-AMPK-, Atg5-, and Atg7-positive and diminished the numbers of p-mTOR-positive cells (Supplementary Fig. S6d), which was consistent with the immuno-blotting assay results (Fig. 1j and Supplementary Fig. S6f). Immuno-blotting assay also suggested that cleaved caspase-8 and cleaved caspase-3 expression was at a higher level in the treatment groups than model. However, there were no significant differences in the survival rate among different groups (Supplementary Fig. S6b). Supplementary Fig. S6g displayed the H&E staining of the major organs including lung, heart, liver, kidney, and spleen. No damage or inflammation appeared in the other two treatment groups, and the body weight of mice remained unchanged during the experiment compared with those of the control group (Supplementary Fig. S6a), altogether confirming the relative safety of trifolirhizin in vivo.

In conclusion, our current study has demonstrated that trifolirhizin induces autophagy through activating AMPK/mTOR pathway and positively contributes to the extrinsic apoptosis in CRC both in vivo and in vitro. The trifolirhizin-mediated autophagic promotion might be a promising therapeutic strategy for CRC.

## Supplementary information

Supplementary Materials for Trifolirhizin induces autophagy-dependent apoptosis in colon cancer via AMPK/mTOR signaling

Supplementary Fig S1-S6

original data of the research
