# Supplementary material for: Trifolirhizin induces autophagy-dependent apoptosis in colon cancer via AMPK/mTOR signaling
Source: Signal Transduct Target Ther. 2020 Aug 27;5:174. doi: 10.1038/s41392-020-00281-w (PMC7452898; doi:10.1038/s41392-020-00281-w)
Supplement: Supplementary file 1 — Supplementary Materials for Trifolirhizin induces autophagy-dependent apoptosis in colon cancer via AMPK/mTOR signaling [file 41392_2020_281_MOESM1_ESM.doc]

Supplementary Materials for

Trifolirhizin induces autophagy-dependent apoptosis in colon cancer via AMPK/mTOR signaling

Dongdong Sun^1,2,3^ ^#^, Weiwei Tao^2,3,4^ ^#^, Feng Zhang^3,4^ ^#^, Weixing Shen^1^, Jiani Tan^1^, Liu Li^1^, Qinghai Meng^3,4^, Yugen Chen^5^, Ye Yang^1^, Haibo Cheng^1,5^

Correspondence to: Haibo Cheng, e-mail: [hbcheng_njucm@163.com](mailto:hbcheng_njucm@163.com); Ye Yang, e-mail: yangye876@sina.com

**Materials and Methods**

### Reagents and antibodies

Trifolirhizin was provided by Sigma-Aldrich. 3-Methyladenine (3-MA), Bafilomycin A1(Baf A1), chloroquine (CQ) and Compound C were used as autophagy inhibitors and were purchased from Sigma-Aldrich (M9281, C6628, 196000). Oxaliplatin (Sigma-Aldrich, O9512) was used as the positive drug in animal experiment. Adenovirus stably expressing mCherry-GFP-LC3B fusion protein (AdmCherry-GFP-LC3B) (Beyotime, C3012) transfected cells to monitor autophagy flux in vitro. Rapamycin, Z-VAD-fmk and Crystal Violet Staining Solution were from Beyotime (S1842, C1202, C0121). AMPK siRNA and ATG5 siRNA was customized from Sangon Biotech.

These antibodies and their producers are as follows. Antibodies against LC3B (ab51520); SQSTM1/p62 (ab91526); GAPDH (ab181602); ubiquitin (ab110601); anti-AMPK alpha 1(Y365) (ab32047); anti-mTOR (Y391) (ab32028); anti-AMPK alpha 1 (phospho S487) (ab131357); anti-mTOR (phospho S2448) (ab109268); anti-cleaved caspase3 (E83-77) (ab32042); anti-cleaved caspase-9 (ab2324); anti-cleaved PARP (E51) (ab32064); anti-lysosomal associated membrane protein 1(LAMP1) (ab25630) and secondary antibody goat anti-rabbit (ab181602) all from Abcam; Cleaved caspase-8 (D384) Polyclonal Antibody (ImmunoWay Biotechnology, YC0011).

### Cell culture

Human colon cancer cells HCT116 and SW480 were supplied by the cell bank of the Chinese Academy of Sciences (TCHu133, TCHu101). The cells were maintained in RPMI Medium 1640 (Jet Bio-Filtration Co., RPM101640) containing 10% heat-inactivated fetal bovine serum (EpiZyme, CY102), 1% streptomycin and penicillin (Beyotime, C0222) at 37 °C in a 5% CO_2_ atmosphere.

### Cell viability assay

The Cells (1.0 × 10^4^) was plated onto 96-Well plates (Jet Bio-Filtration, Guangzhou, China) and cell viability of SW620 and HCT116 cells were measured by CCK-8 assay (Beyotime, C0039), following the manufacturer’s instruction. Cell was treated with different concentrations of trifolirhizin (0, 5, 10, 20, 30 and 40 μg/mL). Cells treated with fresh medium which was added 0.1% DMSO (Sigma-Aldrich, D2650) were used as control. The optical density (OD) was measured at 450 nm by using a microplate reader (ELx800, BioTek, Winooski, VT, USA), the cytotoxic effects of trifolirhizin was calculated using the formula:

### Colony formation assay

Cells were seeded in 6-well culture plates (2,000 cells/well) and treated with different concentration of trifolirhizin. The culture medium was then replaced with fresh medium at a 2 days interval. Next, the cells were fixed using 4% paraformaldehyde and stained for 10 min with crystal violet. The colonies were photographed by a computerized microscope system (Leika, wetzlar, Germany) and colonies formation rate was calculated.

### Western blot assay

Cells at ~80% confluency were seeded in 6-well plates and treated as indicated. The cells were harversted, washed and lysed. Protein lysates of cells or tissues were collected for western blot analysis. Detergent-soluable and -insoluable samples were prepared as described.^1^ After calculating proteins concentration by NanoDrop One (Thermo Scientific, Waltham, MA, USA), the protein lysates were subjected to SDS-PAGE and electrotransfer onto a polyvinylidene difluoride (PVDF) (Millipore, ISEQ00010) membrane following the instructions of commercial manufacturer. Membranes were blocked in 5% (w/v) skimmed-milk and afterwards incubated with a 1:2,000 dilution of primary antibodies at 4°C overnight. Thereafter the membranes were incubated with secondary antibody. Detection of the blots were performed by enhanced chemiluminescent kit (Epizyme, SQ201). The optical densities were analyzed with Bio-Rad image analysis (Bio-Rad, Hercules, CA, USA).

### Cell transfection with Ad-mCherry-GFP-LC3B

Ad-mCherry-GFP-LC3B, an adenovirus expressing mCherry-GFP-LC3B fusion protein, was used to analyze autophagic flux. Cells in logarithmic growth phase were placed in cell culture dishes (5×10^5^/dish; Jet Bio-Filtration, Guangzhou, China) and incubated at 37C in 5% CO_2_. After 6 h, the cells were washed with PBS (pH 7.4; Beyotime, ST476), and Ad-mCherry-GFP-LC3B (20 DOI) was added. After 12 h incubation, trifolirhizin was added and incubated at 37C in 5% CO_2_. The cells were observed under confocal microscopy (Nikon, Melville, NY, USA).

### Apoptosis assays

For the flow cytometry assay, cells were seeded in 6-well culture plates (10^5^ cells/well) and then treated as labeled. Cells were harvested and washed by PBS, after the cells were resuspended in binding buffer, the cell suspensions were mixed with Annexin V-FITC and propidium iodide (PI) (Beyotime, C1062L). After incubation for 15 min, the cell suspensions were analyzed using a FACSCalibur flow cytometer (BD Biosciences, San Jose, CA ,USA). For the terminal deoxynucleotide transferase mediated dUTP nick-end labeling (TUNEL) assay, cells were seeded onto glass coverslips for 24 h and then incubated with or without trifolirhizin for 24 h; the cells were stained using the One Step TUNEL Apoptosis Assay Kit (Beyotime, C1088) in accordance with the manufacturer’s instructions. Apoptosis was identified as the ratios of TUNEL-positive cells per 1,000 DAPI-stained cells. Autophagy-associate apoptosis was detected by Lamp-1 and TUNEL double staining. These samples were then visualized by confocal microscopy.

### Transmission electron microscopy

Transmission electron microscopy was performed according to instructions^2^. Briefly, cells were seeded onto the dish and allowed to proliferate overnight. After that, trifolirhizin-treated cells were fixed with 2.5% glutaraldehyde (Sigma-Aldrich, G6257) in 0.2 M HEPES buffer (pH 7.4; Sigma-Aldrich, 83264) for 30 min, collected and fixed as a pellet at 4 °C. The cell pellets were pooled for the subsequent procedures. The pellets were post-fixed in 1% osmium tetroxide (Sigma-Aldrich, 75632) and 0.5% potassium ferricyanide (Electron Microscopy Sciences, 20150) in cacodylate buffer (Electron Microscopy Sciences, 11650). 1 h later, the samples were embedded in straight resin (Agar Scientific, R1045). After cutting into 60-nm thick sections, the blocks were stained with uranyl acetate (Agar Scientific, R1260A) and lead citrate (Agar Scientific, R1210). Consequently, the samples were visualized under a Zeiss EM 900 transmission electron microscope (Carl Zeiss, [Weimar](https://aws.qa.britannica.com/place/Weimar-Germany), Germany).

### Animal procedures

All animal experimental procedures were carried out in strict accordance with the [Helsinki Declaration of 2013](http://www.tandf.co.uk/journals/authors/KAUP-declaration.pdf). Six-week-old C57BL/6 nude mice (20±3 g) from the Institute of model zoology Laboratory of Nanjing university were were fed standard chow and water. The C57BL/6 mice were subcutaneously injected in the right flanks with the cell suspension (5×10^6^ SW620 cells) and were divided into negetive control, positive drug (oxaliplatin) and trifolirhizin groups, each group was intraperitoneal injection given saline, oxaliplatin (5mg/kg) or trifolirhizin (10 mg/kg) repectively on the day 0, 3, 6, 9, 12, 15, 18 and 21 after inoculating. Serial tumor volume and body weights taken every 3 days during the experiment course, mice were sacrificed on the 21st day to obtain tissue and weighed.

### Histological analysis

Portions of the harvested mice tissue and tumor samples were fixed in 4% paraformaldehyde for 24 h. After dehydration in ethanol, the tissues were embedded in paraffin and sliced into 5 μm. H&E staining slides were stained with hematoxylin-eosin (H&E) kit (Beyotime, C0105) and observed under microscope. The TUNEL reaction was carried out according to the manufacturer’s instructions as mentioned above and was visualized by confocal microscopy. The mean fluorescence intensities of TUNEL per cell and number of DAPI puncta per cell were measured using ImageJ. Immunohistochemistry analysis was also performed on tumor sections that were deparaffinized and incubated with primary antibodies, visualized by an anti-rabbit IgG conjugated with DAB (Beyotime, P0203) and counterstained with Haematoxylin. Images were captured using the microscope. To quantify the level of protein expression, the integrated optical density (IOD) of tumor section was detected automatically by Image-Pro Plus software (Media Cybernetics, Silver Spring, MD, USA).

### Statistical analysis

The statistical analysis was conducted using GraphPad Prism 6.0 software (GraphPad, La Jolla, CA, USA). The data were illustrated as mean ± standard deviation (M ± SD). The differences among groups were assessed using the Student's paired t-test. P values < 0.05 were considered to reflect significant differences.

**References**

1. The selective autophagy substrate p62 activates the stress responsive transcription factor Nrf2 through inactivation of Keap1. *Nature Cell Biology* **12**, 399-403 (2010).

2. Starborg, T. *et al.* Using transmission electron microscopy and 3View to determine collagen fibril size and three-dimensional organization. *Nature Protocols* **8**, 1433-1448 (2013).

**Supplementary figure legends**

**Fig. S1:** **Trifolirhizin-induced autophagy in HCT116 and SW620 cells.** (a) The chemical structure of trifolirhizin. Cells treated with either different concentrations or time durations of trifolirhizin. (b,c) Cellular morphology observed by inverted-phase contrast microscope. (d, e) Immunoblotting assay of LC3B-I, LC3B-II, and SQSTM1 protein expression. GADPH was used as loading control. (f) The expression of p62, LC-3B, and ubiquitin in the detergent-soluble or -insoluble fractions of HCT116 and SW620 cells was analyzed by western blotting. (g) Autophagosome quantification of the image taken by scanning electron microscope (Fig 1b). (h) Cells were mixed with Ad-mCherry-GFP-LC3B and then incubated with trifolirhizin for 6 h, and the mcherry (red) and GFP (green) were analyzed by a [laser](../../../../C:/Users/%25E8%2589%25AF%25E7%25BA%25AC/AppData/Local/youdao/dict/Application/8.6.2.0/resultui/html/index.html#/javascript:;) [scanning](../../../../C:/Users/%25E8%2589%25AF%25E7%25BA%25AC/AppData/Local/youdao/dict/Application/8.6.2.0/resultui/html/index.html#/javascript:;) [confocal](../../../../C:/Users/%25E8%2589%25AF%25E7%25BA%25AC/AppData/Local/youdao/dict/Application/8.6.2.0/resultui/html/index.html#/javascript:;) [microscope](../../../../C:/Users/%25E8%2589%25AF%25E7%25BA%25AC/AppData/Local/youdao/dict/Application/8.6.2.0/resultui/html/index.html#/javascript:;) (LSCM), scale bar: 5 μm. The results were presented as the M±SD (n≥3), *P<0.05 and **P<0.01 versus control.

**Fig. S2:** **Analysis of autophagy flux.** HCT116 and SW620 cells were treated with 20μM trifolirhizin in the presence or absence of 10mM 3-MA. (a) Cellular morphology observed by inverted-phase contrast microscope. (b) Expressions of autophagy markers LC3-Ⅰ, LC3-Ⅱand SQSTM1 detected by Western blot assay. Cells were treated with 20μM trifolirhizin in the presence or absence of 20μM CQ or 10nM BafA1. (c) Cellular morphology observed by inverted-phase contrast microscope. (d, e) Expressions of autophagy markers LC3-Ⅰ, LC3-Ⅱand SQSTM1 detected by Western blot assay. The results were presented as the M±SD (n=3), **P<0.01 versus control and ^#^P<0.05 ^##^P<0.01 versus Trifolirhizin.

**Fig. S3:** AMPK/mTOR pathway was involved in trifolirhizin-induced autophagy in CRC cells. HCT116 and SW620 cells were treated with either different concentrations or time durations of trifolirhizin. (a, b) Western blot assay of p-AMPK, AMPK, p-mTOR, mTOR protein expression. Cells were pre-treated with AMPK inhibitor, Compound C (10 mM) or AMPK siRNA, followed by trifolirhizin(20μM) treatment for 12 h. (c, d) Western blot analysis of p-mTOR, mTOR, LC3B-I, LC3B-II, SQSTM1. (e, f) Cells were mixed with Ad-mCherry-GFP-LC3B, the mCherry (red) and GFP (green) were analyzed by LSCM and were quantified by ImageJ software. (g, h) Effects of the mTOR inhibitor, Rapamycin, were clrarified by autophagy flux analysis. The results were represented as the M±SD (n≥3), *P<0.05 **P<0.01 versus 0 μM/ 0 hour/ control and ^#^P<0.05 ^##^P<0.01 versus Trifolirhizin.

**Fig. S4: Trifolirhizin induced apoptosis of CRC cells through caspase dependent pathway.** (a) Effects of trifolirhizin on cell viability of HCT116 and SW480 cells. The cells were exposed to different concentrations of trifolirhizin for 48 h, and the cell viability was measured by CCK-8 assay. (b) Effects of trifolirhizin on the colony formation. (c) Early apoptosis of the cell was analyzed by flow cytometry with AnnexinⅤ-FITC/PI staining. (d) Late apoptosis was measured by TUNEL assay. (e, f) Expressions of Cleaved-caspase-9, Cleaved-caspase-8, Cleaved-caspase-3, Cleaved-PARP and Cytochrome C were determined by western blot. Cells were treated with Z-VAD-fmk in the absence or presence of 20μM trifolirhizin, followed by (g) Colony formation assay and (h) AnnexinⅤ-FITC/PI assay. The results were represented as the M±SD (n≥3), **P<0.01 versus 0μM/ control and ^##^P<0.01 versus Trifolirhizin.

**Fig. S5: Autophagy induced by trifolirhizin contributes to apoptosis of CRC cells.** HCT116 and SW620 cells were pre-treated with Autophagy inhibitors, CQ (20 μM), Compound C (10 μM), ATG5 siRNA, followed by trifolirhizin (20 μM) treatment for 48h. Colony formation assay (a, d), AnnexinⅤ-FITC/PI assay (b, e), Western-blot assays of cleaved-caspase-8, cleaved-caspase-3, cleaved-PARP (c, f) were performed. (g) Autophagy-associated apoptosis was detected by Lamp-1 (green) and TUNEL (red) co-staining. The cell nuclei were counterstained with DAPI (blue). Scale bar: 20 μm. The results were represented as the M±SD (n≥3), **P<0.01 versus control and ^##^P<0.01 versus Trifolirhizin.

**Figure S6: Trifolirhizin inhibited tumor growth and induced autophagy in vivo.** Tumor bearing mice were treated with trifolirhizin, Oxaliplatin (positive drug) or with vehicle control. (a) Mice body weight was checked every 3 day. (b) Survival rate was monitored within 21 days. H&E staining (c) and TUNEL staining (d) were conducted on tumor sections harvested from mice. (e) The expression of p-AMPK, p-mTOR, Atg5, Atg7, Cleaved-caspase-8 and Cleaved-caspase-3 was determined by [immumohistochemical](../../../../C:/Users/%25E8%2589%25AF%25E7%25BA%25AC/AppData/Local/youdao/dict/Application/8.6.2.0/resultui/html/index.html#/javascript:;) [staining](../../../../C:/Users/%25E8%2589%25AF%25E7%25BA%25AC/AppData/Local/youdao/dict/Application/8.6.2.0/resultui/html/index.html#/javascript:;). (f) Western blot assays of p-AMPK, AMPK, p-mTOR, mTOR, Atg5, Atg7, Cleaved-caspase-8 and Cleaved-caspase-3. (g) H&E staining of lung, kidney, liver and heart tissue samples obtained from the indicated groups of mice. The results were represented as the M±SD (n≥3), **P<0.01 versus model, scale bar: 50 μm.
