# Supplementary figures and images for: Trifolirhizin induces autophagy-dependent apoptosis in colon cancer via AMPK/mTOR signaling
Source: Signal Transduct Target Ther. 2020 Aug 27;5:174. doi: 10.1038/s41392-020-00281-w (PMC7452898; doi:10.1038/s41392-020-00281-w)

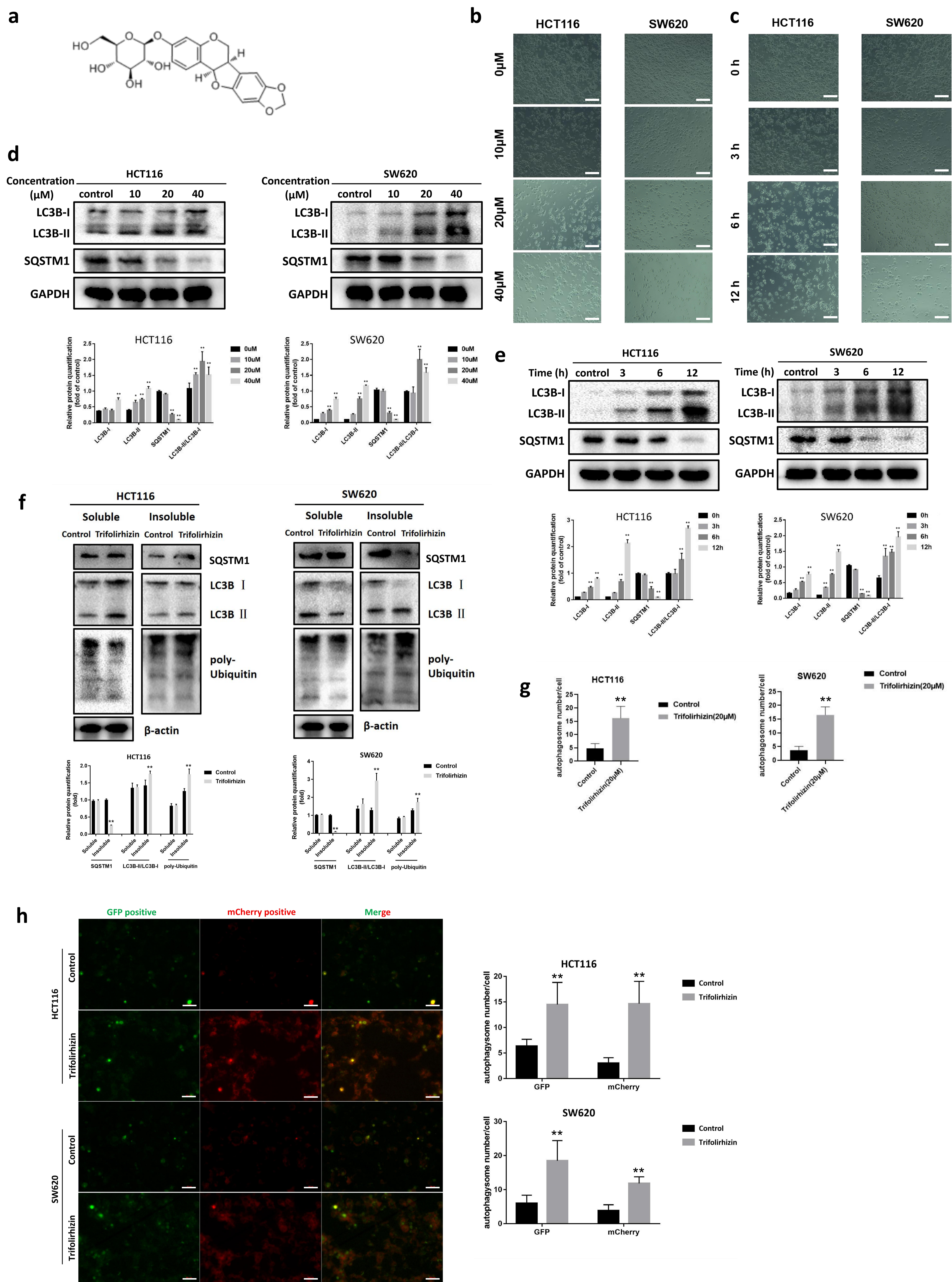

Figure S1

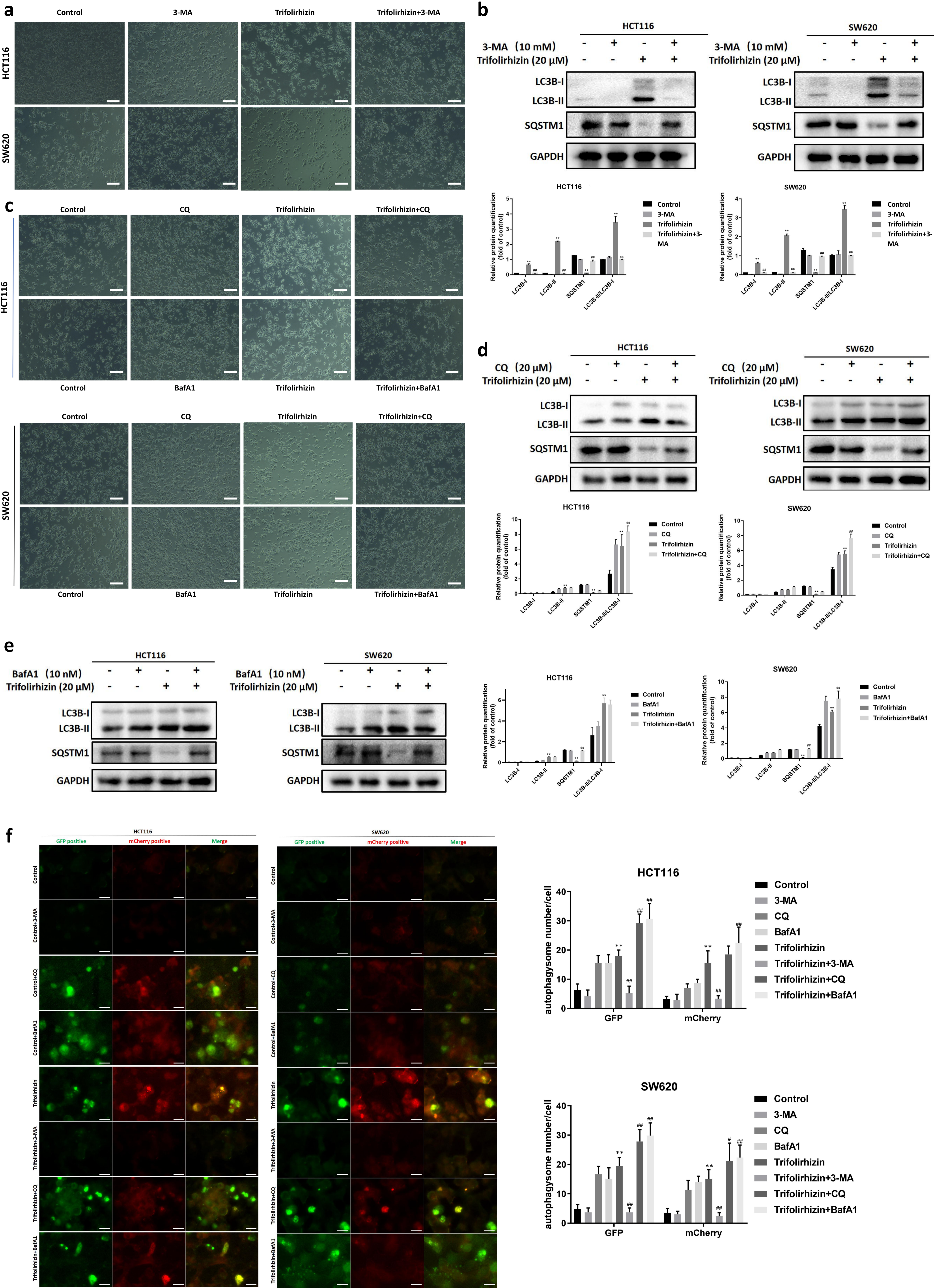

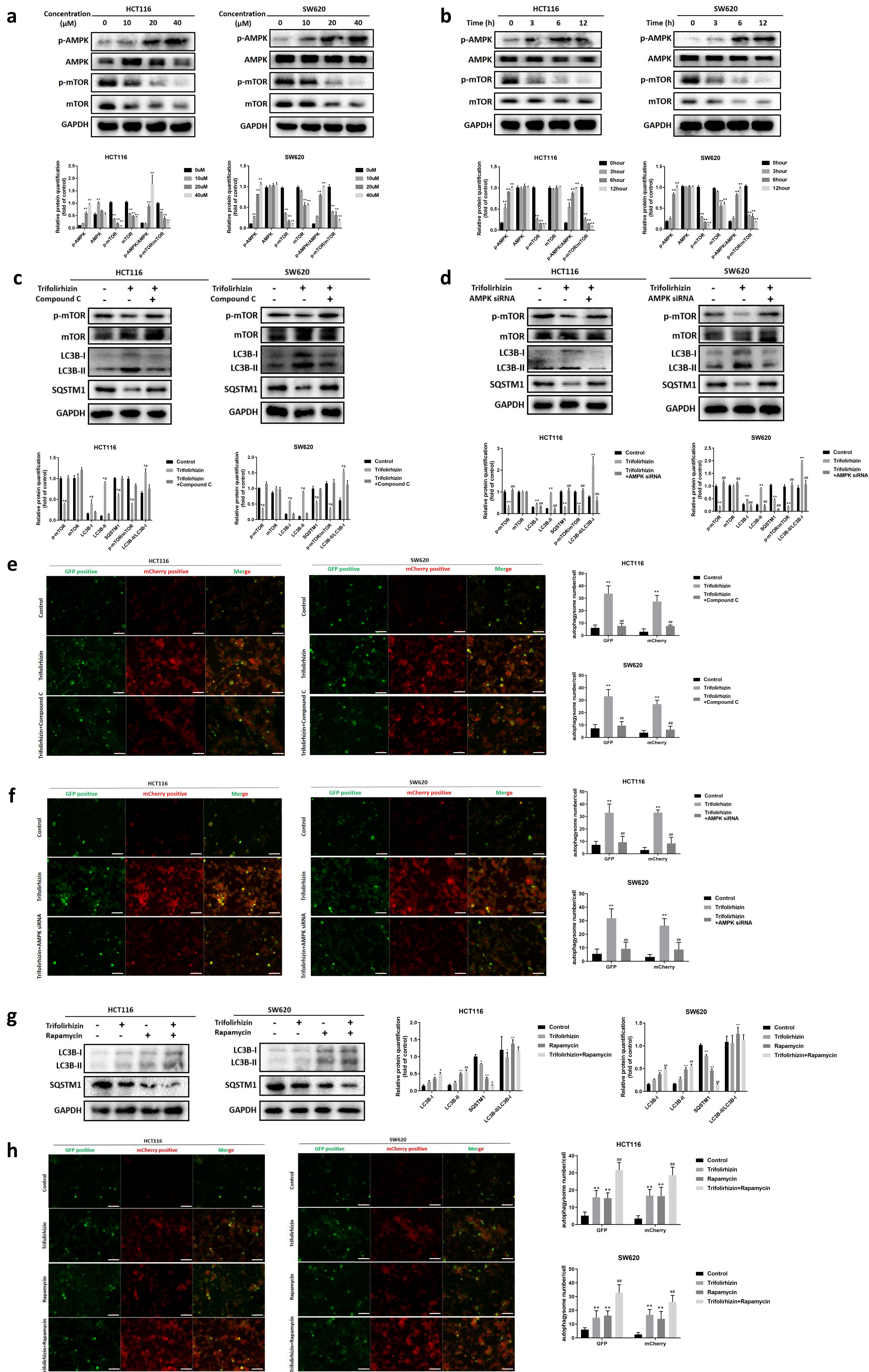

Figure 3



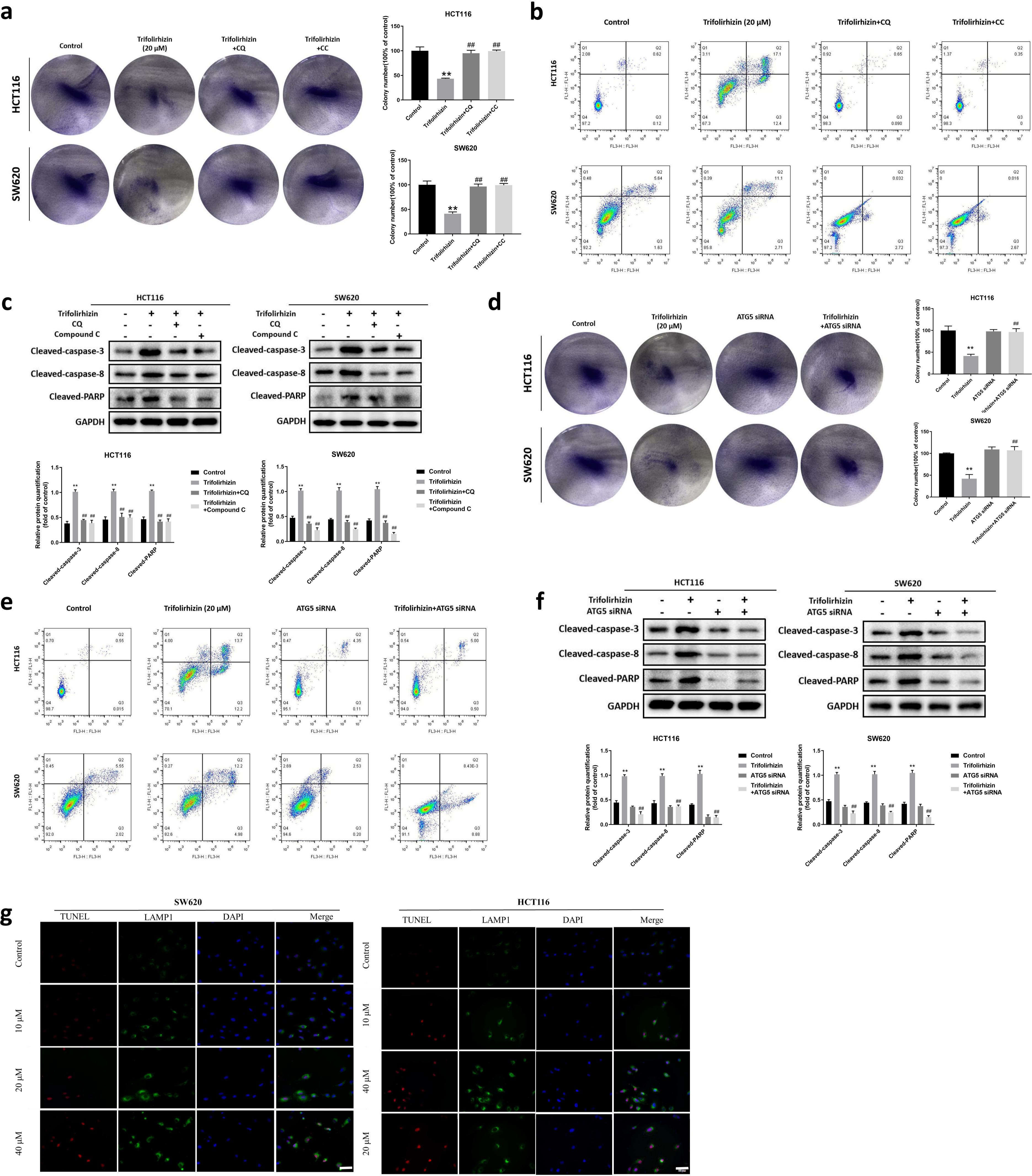

Figure S5

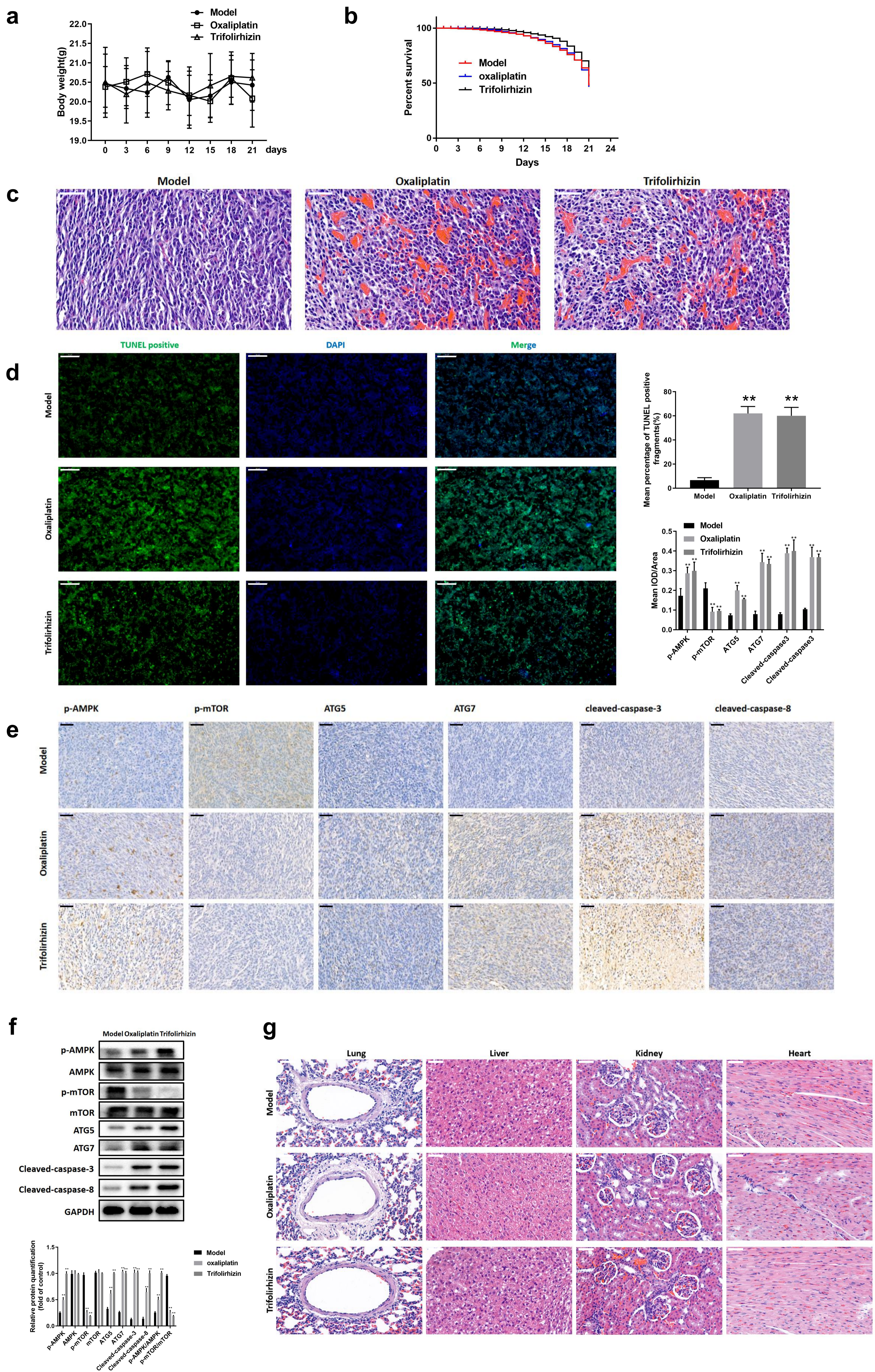

Figure S6

Supplement: Supplementary file 2 — Supplementary Fig S1-S6 [file 41392_2020_281_MOESM2_ESM.pdf]
